# Hyaline-vascular type unicentric Castleman disease with dysplastic follicular dendritic cell proliferative lesions: a case report

**DOI:** 10.1093/jscr/rjad536

**Published:** 2023-09-27

**Authors:** Daisuke Horikawa, Ryotaro Shimazaki, Kazuya Manabe, Kentaro Ichimura, Kei Ishibashi, Yasutomo Fukasaku, Takahisa Ishikawa, Yasuyuki Koshizuka, Taiichiro Shibaki, Naoyuki Yanagida, Hiromitsu Akabane, Hideki Yokoo, Yasuo Sumi

**Affiliations:** Department of Surgery, Furano Kyokai Hospital, Furano, Hokkaido 076-8765, Japan; Department of Surgery, Asahikawa Kosei Hospital, Asahikawa, Hokkaido 078-8211, Japan; Department of Surgery, Asahikawa Kosei Hospital, Asahikawa, Hokkaido 078-8211, Japan; Department of Surgery, Asahikawa Kosei Hospital, Asahikawa, Hokkaido 078-8211, Japan; Department of Surgery, Asahikawa Kosei Hospital, Asahikawa, Hokkaido 078-8211, Japan; Department of Surgery, Asahikawa Kosei Hospital, Asahikawa, Hokkaido 078-8211, Japan; Department of Surgery, Asahikawa Kosei Hospital, Asahikawa, Hokkaido 078-8211, Japan; Department of Surgery, Asahikawa Kosei Hospital, Asahikawa, Hokkaido 078-8211, Japan; Department of Surgery, Asahikawa Kosei Hospital, Asahikawa, Hokkaido 078-8211, Japan; Department of Surgery, Asahikawa Kosei Hospital, Asahikawa, Hokkaido 078-8211, Japan; Department of Surgery, Asahikawa Kosei Hospital, Asahikawa, Hokkaido 078-8211, Japan; Department of Hepatobiliary, Pancreatic and Transplant Surgery, Asahikawa Medical University, Asahikawa, Hokkaido 078-8510, Japan; Department of Digestive Surgery, Asahikawa Medical University, Asahikawa, Hokkaido 078-8510, Japan

**Keywords:** Castleman disease, Castleman’s disease, follicular dendritic cell, unicentric, hyaline-vascular

## Abstract

Castleman disease (CD) is a rare lymphoproliferative disease. Hyaline-vascular type unicentric CD has a good prognosis if completely resected during surgery. However, follicular dendritic cell proliferative lesions have the potential for recurrence and metastasis. A 22-year-old man was referred to our hospital with the chief complaint of nausea and vomiting. These symptoms were caused by a right mesocolonic tumor pushing the duodenum. The patient underwent laparoscopic tumorectomy and complete surgical excision. The postoperative course was uneventful, with no complications. Pathological examination confirmed that the tumor was an enlarged lymph node, typical of hyaline vascular-type CD; however, follicular dendritic cell proliferative lesions were noted. We report a rare case of hyaline-vascular-type CD with follicular dendritic cell proliferative lesions associated with malignancy, as limited case reports exist on this particular disease.

## Introduction

Castleman disease (CD) is a rare lymphoproliferative disease that was first reported by Castleman in 1954 [1, 2]. Currently, this disease can be classified as unicentric CD (UCD) or multicentric CD (MCD). Patients with UCD exhibit a good prognosis if they undergo complete surgical excision. In contrast, a previous case report showed that UCD with proliferative follicular dendritic cell (FDC) lesions had the potential for metastasis and recurrence, which developed at various intervals from the initial diagnosis, with the longest recurrence or metastasis occurring after 11 years [3]. Due to the scarcity of case reports on this disease, we report a rare case of UCD with dysplastic FDC proliferative lesions.

## Case report

**Figure 1:**
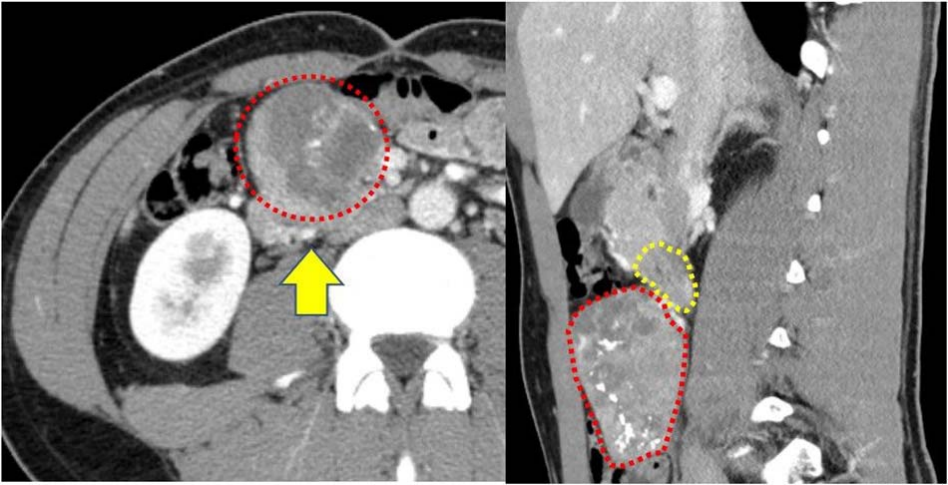
(a) Contrast-enhanced computed tomography reveals a well-defined heterogeneous enhancement mass with calcification (red dotted box); the mass is present on the ventral ileocolic vessels (yellow arrow); (b) the transverse duodenum (yellow dotted box) is pushed by this tumor (red dotted box).

**Figure 2:**
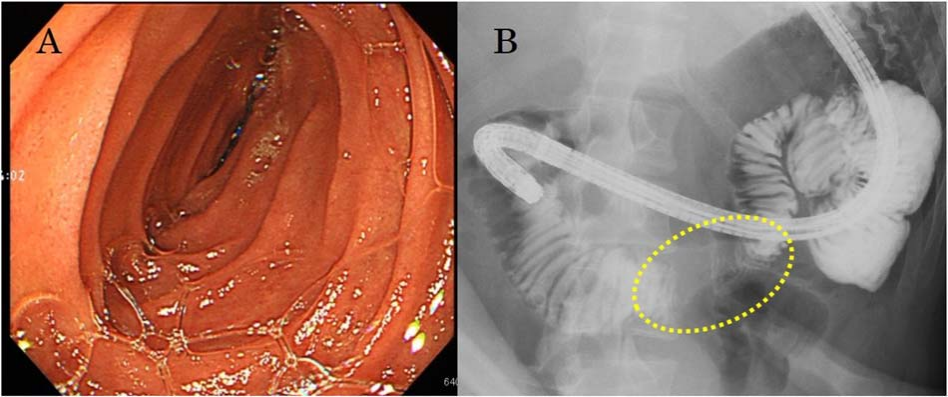
(a) Esophagogastroduodenoscopy reveals no lesion in the duodenal lumen, but the transverse duodenum is pushed by the mass from outside the lumen; (b) upper gastrointestinal examination illustrates that the transverse duodenum is pressed by the mass palpable outside the body of the patient.

A 22-year-old man was referred to our department with the chief complaint of nausea and vomiting. An abdominal examination revealed a mass with poor mobility and elastic hardness on the right side of the navel. Further evaluations using abdominal ultrasonography revealed an ~83 × 51 × 49 mm well-defined, oval, hypoechoic, and heterogeneously enhanced mass with a strong echo. A contrast-enhanced computed tomography scan showed a well-defined, heterogeneous enhancement with calcification. The tumor pushing the transverse part of the duodenum was present on the ventral ileocolic vessels and measured ~6 cm (Fig. 1). Magnetic resonance imaging was performed to confirm invasion of other organs. All of the above examinations suggested that the tumor had not invaded other organs, especially the duodenum. Esophagogastroduodenoscopy revealed no lesions in the duodenal lumen. However, the duodenum was pushed by a mass outside the lumen (Fig. 2).

**Figure 3:**
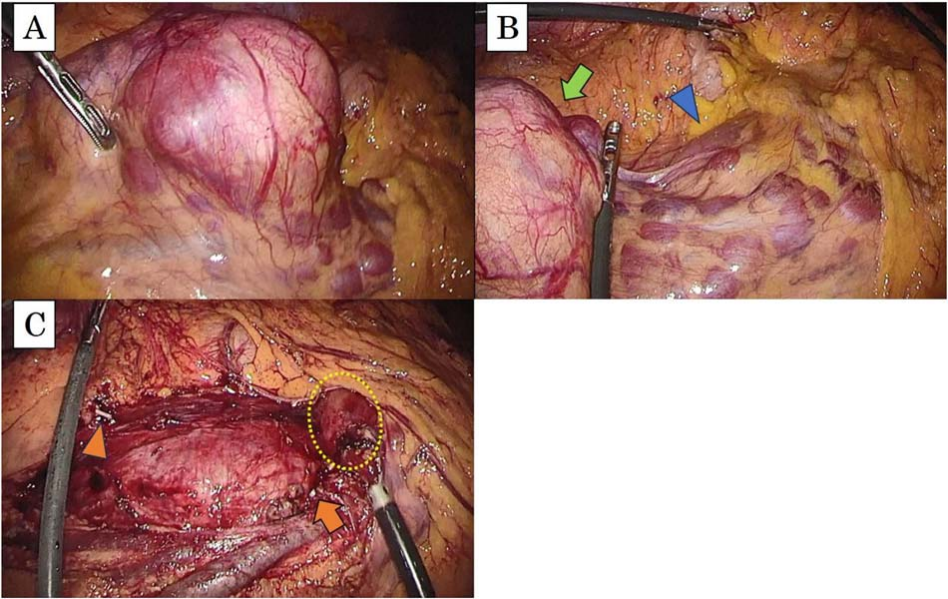
(a) The tumor is present in the right mesocolon; (b) there is no invasion from the tumor (arrow) to the duodenum (arrowhead); (c) this intraoperative image, which has resected the tumor, shows the periphery of ileocolic vessels (arrow head) and the root of the vessels (arrow); the duodenum is not damaged (dotted box).

Since we could not obtain a definite diagnosis preoperatively, the patient underwent a laparoscopic tumorectomy for diagnosis and therapy. The tumor was localized around the ileocolic vessels without duodenal invasion and completely resected with the ileocolic vessels (Fig. 3). The postoperative course was uneventful, and there was no recurrence within the first year and 6 months.

**Figure 4:**
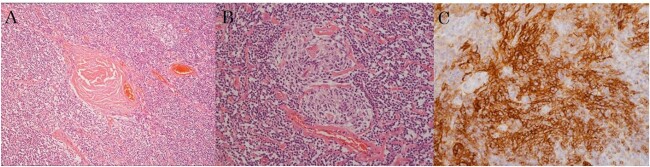
Pathologic examination based on hematoxylin and eosin staining shows the following: (a) microvascular proliferation and angio-sclerosis, (b) atrophy of the germinal center, (c) FDC with CD21-positive proliferative lesion.

Pathologically, the tumor was identified as an enlarged lymph node measuring 8 × 6 × 5 cm. It exhibited characteristics typical of hyaline vascular UCD. However, dysplastic FDC proliferative lesions positive for CD21 were also observed, with the largest lesion measuring 5 mm (Fig. 4).

## Discussion

CD is a rare lymphoproliferative disorder with several subtypes. It can be categorized as either UCD or MCD. MCD is often characterized by systemic symptoms, such as anemia, fever, and malaise and has various subtypes, including those associated with human herpes virus type 8 (HHV-8); polyneuropathy, organomegaly, endocrinopathy, monoclonal gammopathy, and skin changes (POEMS) syndrome; and idiopathic cases. The treatment of idiopathic MCD involves the use of immunosuppressive drugs, such as steroids and IL-6 inhibitors. MCD-associated HHV-8 requires rituximab and liposomal doxorubicin, while HIV-positive patients require combination antiretroviral therapy. In the case of POEMS syndrome, patients require therapy for multiple myeloma.

In contrast, UCD rarely presents with symptoms, with occasional fever and malaise; therefore, histopathological examination is indispensable for a formal diagnosis. Complete surgical excision is recommended for the treatment of UCD, which has a good prognosis. In a study by Talat *et al*. [4], complete surgical excision showed that the 3-year disease-free survival rate for patients with hyaline vascular CD was 92.5%. In another study, Talat *et al*. [5] reported that complete surgical excision had better outcomes than diagnostic resection during a 10-year follow-up (mortality rate: 3.8% vs. 17.6%). In this context, the primary therapy for UCD is surgery, and complete resection of the tumor is crucial. If complete resection is not possible, radiation and neoadjuvant therapy should be considered. These therapies have the possibility to cause shrinkage of the unresectable tumors [6–8].

However, in UCD, if FDC proliferative lesions are present, metastasis and recurrence may occur. Lin *et al*. [3] reported that three-fifths of UCD cases had FDC proliferative lesions with recurrence or metastatic dissemination. Surprisingly, the longest recurrence duration was 11 years. Although the others were disease-free, the follow-up period was very short at 2 and 6 months.

In our case, the patient only experienced nausea and vomiting, and the duodenum was compressed by the giant mass. Clinical diagnosis was difficult due to the lack of specific symptoms; hence, we performed a laparoscopic tumorectomy for both diagnosis and therapy. The patient underwent complete surgical excision and showed no evidence of recurrence or metastasis after a follow-up period of 18 months post-surgery. However, as mentioned above, UCD with FDC proliferative lesions has a possibility of malignancy, and the longest term of recurrence was 11 years. Therefore, the long-term follow-up of this patient is essential.

To the best of our knowledge, there is currently limited data on UCD with FDC proliferative lesions. Thus, more case reports are required to elucidate the mechanisms through which FDC proliferation is associated with recurrence and metastasis.

## References

[1] Castleman B, Towne VW. CASE records of Massachusetts General Hospital Weekly Clinicopathological Exercises: Case 40011. N Engl J Med 1954;251:396–400. 13194083 10.1056/NEJM195409022511008

[2] Castleman B, Iverson L, Menendez VP. Localized mediastinal lymph-node hyperplasia resembling thymoma. Cancer 1956;9:822–30. 13356266 10.1002/1097-0142(195607/08)9:4<822::aid-cncr2820090430>3.0.co;2-4

[3] Lin O, Frizzera G. Angiomyoid and follicular dendritic cell proliferative lesions in Castleman’s disease of hyaline-vascular type: a study of 10 cases. Am J Surg Pathol 1997;21:1295–306. 9351567 10.1097/00000478-199711000-00004

[4] Talat N, Schulte KM. Castleman’s disease: systematic analysis of 416 patients from the literature. Oncologist 2011;16:1316–24. 21765191 10.1634/theoncologist.2011-0075 PMC3228165

[5] Talat N, Belgaumkar AP, Schulte KM. Surgery in Castleman’s disease. Ann Surg 2012;255:677–84. 22367441 10.1097/SLA.0b013e318249dcdc

[6] Boutboul D, Fadlallah J, Chawki S, et al. Treatment and outcome of Unicentric Castleman disease: a retrospective analysis of 71 cases. Br J Haematol 2019;186:269–73. 31016730 10.1111/bjh.15921

[7] Kyu Noh O, Lee SW, Lee JW, et al. Cases report of unicentric castleman’s disease: revisit of radiotherapy role. Radiat Oncol J 2013;31:48–54. 23620869 10.3857/roj.2013.31.1.48 PMC3633231

[8] Chan KL, Lade S, Prince HM, Harrison SJ. Update and new approaches in the treatment of Castleman disease. J Blood Med 2016;7:145–58. 27536166 10.2147/JBM.S60514 PMC4976903

